# Passive leg raising: five rules, not a drop of fluid!

**DOI:** 10.1186/s13054-014-0708-5

**Published:** 2015-01-14

**Authors:** Xavier Monnet, Jean-Louis Teboul

**Affiliations:** Service de réanimation médicale, Hôpital de Bicêtre, Hôpitaux Universitaires Paris-Sud, 78, rue du Général Leclerc, Le Kremlin-Bicêtre, Paris, F-94270 France; Faculté de médecine Paris-Sud, Université Paris-Sud, EA4533, Le Kremlin-Bicêtre, Paris, F-94270 France

In acute circulatory failure, passive leg raising (PLR) is a test that predicts whether cardiac output will increase with volume expansion [1]. By transferring a volume of around 300 mL of venous blood [2] from the lower body toward the right heart, PLR mimics a fluid challenge. However, no fluid is infused and the hemodynamic effects are rapidly reversible [1,3], thereby avoiding the risks of fluid overload. This test has the advantage of remaining reliable in conditions in which indices of fluid responsiveness that are based on the respiratory variations of stroke volume cannot be used [1], like spontaneous breathing, arrhythmias, low tidal volume ventilation, and low lung compliance.

The method for performing PLR is of the utmost importance because it fundamentally affects its hemodynamic effects and reliability. In practice, five rules should be followed.

First, PLR should start from the semi-recumbent and not the supine position (Figure 1). Adding trunk lowering to leg raising should mobilize venous blood from the large splanchnic compartment, thus magnifying the increasing effects of leg elevation on cardiac preload [2] and increasing the test’s sensitivity. A study that did not comply with this rule misleadingly reported a poor reliability of PLR [4].

**Figure 1:**
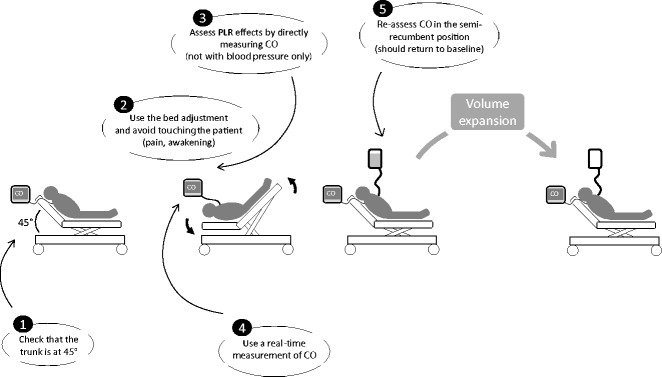
**The best method for passive leg raising, indicating the five rules to be followed.** CO, cardiac output; PLR, passive leg raising.

Second, the PLR effects must be assessed by a direct measurement of cardiac output and not by the simple measurement of blood pressure. Indeed, reliability of PLR is poorer when assessed by using arterial pulse pressure compared with cardiac output [1,5]. Although the peripheral arterial pulse pressure is positively correlated with stroke volume, it also depends on arterial compliance and pulse wave amplification. The latter phenomenon could be altered during PLR, impeding the use of pulse pressure as a surrogate of stroke volume to assess PLR effects.

Third, the technique used to measure cardiac output during PLR must be able to detect short-term and transient changes since the PLR effects may vanish after 1 minute [1]. Techniques monitoring cardiac output in ‘real time’, such as arterial pulse contour analysis, echocardiography, esophageal Doppler, or contour analysis of the volume clamp-derived arterial pressure, can be used [6]. Conflicting results have been reported for bioreactance [7,8]. The hemodynamic response to PLR can even be assessed by the changes in end-tidal exhaled carbon dioxide, which reflect the changes in cardiac output in the case of constant minute ventilation [5].

Fourth, cardiac output must be measured not only before and during PLR but also after PLR when the patient has been moved back to the semi-recumbent position, in order to check that it returns to its baseline (Figure 1). Indeed, in unstable patients, cardiac output changes during PLR could result from spontaneous variations inherent to the disease and not from cardiac preload changes.

Fifth, pain, cough, discomfort, and awakening could provoke adrenergic stimulation, resulting in mistaken interpretation of cardiac output changes. Some simple precautions must be taken to avoid these confounding factors (Figure 1). PLR must be performed by adjusting the bed and not by manually raising the patient’s legs. Bronchial secretions must be carefully aspirated before PLR. If awake, the patient should be informed of what the test involves. A misleading sympathetic stimulation can be suspected if PLR is accompanied by a significant increase in heart rate, which normally should not occur.

It has been suggested that PLR is unreliable in the case of intra-abdominal hypertension [9]. The increased abdominal weight was hypothesized to squeeze the inferior vena cava in the raised-leg position [10]. Nevertheless, the single study investigating this issue did not confirm the hypothesis since intra-abdominal pressure was not measured during PLR [9]. Furthermore, one could hypothesize that PLR reduces rather than increases the intra-abdominal pressure by relieving the weight of the diaphragm on the abdominal cavity.

Provided that these simple rules are followed, the PLR test reliably predicts preload responsiveness [11]. Because it has no side effects, PLR should be considered as a replacement for the classic fluid challenge [12]. The main drawback of the fluid challenge is that, if it is negative, fluid has nonetheless been irreversibly administered to the patient. Repeated fluid challenges therefore can lead to fluid overload. In this regard, PLR is an attractive method of challenging preload without administering one drop of fluid. Importantly, it should be remembered that detection of preload responsiveness by a positive PLR test should not routinely lead to fluid administration. Indeed, the decision to administer fluid must always be made individually on the basis of the mandatory presence of the three following situations: hemodynamic instability or signs of circulatory shock (or both), preload responsiveness (positive PLR test), and limited risks of fluid overload. Also, a negative PLR test should contribute mainly to the decision to stop or discontinue fluid infusion, in order to avoid fluid overload, suggesting that hemodynamic instability should be corrected by means other than fluid administration.

## Abbreviation

PLR: Passive leg raising
